# Preventive Effect of *Zea mays* L. (Purple Waxy Corn) on Experimental Diabetic Cataract

**DOI:** 10.1155/2014/507435

**Published:** 2014-01-16

**Authors:** Paphaphat Thiraphatthanavong, Jintanaporn Wattanathorn, Supaporn Muchimapura, Wipawee Thukham-mee, Panakaporn Wannanon, Terdthai Tong-un, Bhalang Suriharn, Kamol Lertrat

**Affiliations:** ^1^Department of Physiology and Graduate School (Neuroscience Program), Faculty of Medicine, Khon Kaen University, Khon Kaen 40002, Thailand; ^2^Integrative Complementary Alternative Medicine Research and Development Center, Khon Kaen University, Khon Kaen 40002, Thailand; ^3^Department of Physiology, Faculty of Medicine, Khon Kaen University, Khon Kaen 40002, Thailand; ^4^Faculty of Agriculture, Khon Kaen University, Khon Kaen 40002, Thailand

## Abstract

Recently, substances possessing antioxidant can prevent cataractogenesis of diabetic cataract. Therefore, this study was carried out to determine the anticataract effect of *Zea mays* L. (purple waxy corn), a flavonoids rich plant, in experimental diabetic cataract. Enucleated rat lenses were incubated in artificial aqueous humor containing 55 mM glucose with various concentrations of *Zea mays* L. (purple waxy corn) ranging between 2, 10, and 50 mg/mL at room temperature for 72 h. At the end of the incubation period, the evaluation of lens opacification, MDA level, and the activities of SOD, CAT, GPx, and AR in lens were performed. The results showed that both medium and high doses of extract decreased lens opacity together with the decreased MDA level. In addition, medium dose of extract increased GPx activity while the high dose decreased AR activity. No other significant changes were observed. The purple waxy corn seeds extract is the potential candidate to protect against diabetic cataract. The mechanism of action may occur via the decreased oxidative stress and the suppression of AR. However, further research in vivo is still essential.

## 1. Introduction 

Cataract, a visual impairment induced by optical dysfunction of crystallin lens, is an important complication of diabetic patients. It has been recognized as a major cause of blindness in developed and developing countries [1]. The prevalence is continually increased together with the increased amount of diabetic patients [2]. It also produces a great impact on annual health care budget [3]. Therefore, the preventive strategy against diabetic cataract in diabetic patients is regarded as a challenge in this decade. Oxidative stress has been reported to be associated with diabetes mellitus and its complications [4]. Under normal circumstance, the toxic effect of oxidative stress is neutralized in lens by both enzymatic and nonenzymatic antioxidants. However, the activities of superoxide dismutase (SOD) and catalase (CAT), the main important enzymatic antioxidants in lens of diabetic cataract, are decreased. The decrease of antioxidant enzymes mentioned earlier appears to play a pivotal role on the elevation of oxidative stress and cataractogenesis of diabetic cataract [5]. Several lines of evidence have demonstrated that flavonoids, the important phenolic compounds in fruits and vegetables, exert the protective effect against cataractogenesis of diabetic cataract [6, 7].

Recent evidence also shows that aldose reductase (AR), a key enzyme in polyol pathway, also plays the crucial role on the cataractogenesis in diabetic condition [8]. The suppression of aldose reductase activity either by synthetic compounds or by natural flavonoids can protect against cataractogenesis of diabetic cataract [9]. Based on the crucial role of oxidative stress and aldose reductase on cataractogenesis of diabetic cataract and the beneficial effect of flavonoids, the prophylactic effect of flavonoids rich substance against diabetic cataract has gained attention.

*Zea mays* L. (purple waxy corn), a plant in a family of Poaceae, is an important source of anthocyanins. Previous studies have demonstrated that the main ingredient of water extract is anthocyanin whereas the main ingredients in non-polar solvent are phenolic acids such as p-coumaric, vanillic acid, protocatechuic acids, flavonoids such as quercetin and a hesperitin derivatives [10]. Consumption of hydroalcoholic extract of purple corn is safe up to 3.5 g·kg^−1^ [11]. In addition, flavonoids including quercetin, the active ingredient in *Zea mays* L. (purple waxy corn), also suppress aldose reductase inhibitory activity and effectively delay the cataractogenesis in diabetic condition [12, 13]. Based on the beneficial effects of anthocyanins and quercetin mentioned earlier, we hypothesized that hydroalcoholic extract of purple waxy corn, the substance which is rich in anthocyanins and flavonoids including quercetin, could mitigate the cataractogenesis in diabetic condition. Unfortunately, no data is available until now. Therefore, in this study we aimed to determine the anticataract effect of hydroalcoholic extract of purple waxy corn in experimental diabetic cataract.

## 2. Materials and Methods 

### 2.1. Plant Material and Extract Preparation

Dried seeds of purple waxy corn or *Zea mays* L. (KKU open pollinated cultivar) during September 2012 were used in this study. Plant materials were authenticated by Associate Kamol Lertrat and Dr. Bhalang Suriharn, Faculty of Agriculture, Khon Kaen University, Khon Kaen, Thailand. After being authenticated, the herbarium specimen was kept at the Integrative Complementary Alternative Medicine, Khon Kaen University (voucher specimen 2012001). Dried seeds of purple waxy corn had been subjected to extraction with 50% hydro-alcoholic solvent at room temperature with a ratio of 2 : 5 (weight : volume) for 3 days. The extract was concentrated by using lyophilization and at 4°C until used for further study. The yielding extract was 5.72% and contained total phenolic compounds and anthocyanins at concentrations of 21.22 ± 0.08 mg/L GAE/mg extract and 2371.24 ± 0.08 mg/L cyanidin-3-glucoside equivalents/mg extract, respectively.

### 2.2. Experimental Design

Male Wistar rats, weighing 280–300 g, were used in this study. The animals were maintained and treated in accordance with the guideline and approval of the Ethical Committee on Animals Experiments of Khon Kaen University (AEKKU 98/2555). When the rats were sacrificed, the eyes were enucleated via posterior approach to avoid the damage. The isolated transparent lenses were incubated in artificial aqueous humor (NaCl 140 mM, KCl 5 mM, MgCl_2_ 2 mM, NaHCO_3_ 0.5 mM, Na_2_HPO_4_ 0.5 mM, CaCl_2_ 0.4 mM, and glucose 5.5 mM) for 72 hours at room temperature. The pH is maintained at 7.8 throughout the incubation period. To prevent microbial contamination, strict aseptic techniques were performed and antibiotic drugs including penicillin 32 mg and streptomycin 250 mg were added to the culture media. In addition, glucose at concentration of 55 mM was added to the media in order to develop the model of diabetic cataract. All lenses were divided into various groups as follows; (1) normal lens (glucose 5 mM); (2) diabetic cataract (glucose 55 mM): glucose at concentration of 55 mM was added to all lens to mimic diabetic cataract; (3) diabetic cataract + quercetin (2.4 *μ*g/mL): this group was served as positive control and all lenses in this group were subjected to high concentration of glucose and quercetin, a phenolic compound which previously demonstrated anti-cataract effect; (4)–(6) diabetic cataract + various doses of *Zea mays* L. extract ranging between 2, 10, and 50 mg/mL respectively; all rats in these groups were exposed to high glucose concentration [14] and received *Zea mays* L. extract at doses of 2, 10, and 50 mg/mL, respectively.

### 2.3. Evaluation of Lens Opacity

After 72 hours of incubation, lenses were observed for opacity and photographs were taken by placing the lens on the paper with posterior surface touching the paper, and the number of visible clear squares was observed through the lens during the evaluation of lens opacity [15].

### 2.4. Homogenate Preparation

After 72 hours of incubation, homogenate of lens was prepared in Tris buffer (0.23 M, pH 7.8) containing 0.25 × 10^−3^ Sub M EDTA and homogenate adjusted to 10% w/v. The homogenate was centrifuged at 10,000 g at 4°C for 1 hour and the supernatant was used for the estimation of biochemical parameters [15].

### 2.5. Biochemical Parameters

#### 2.5.1. Determination of Total Phenolic Compounds

A total polyphenol compound was measured using Folin-Ciocalteu colorimetric method described previously by a slightly modified method of Quettier-Deleu et al. (2000) [16]. Purple waxy corn extracts (20 *μ*L) were mixed with 0.2 mL of Folin-Ciocalteu reagent and 2 mL of distilled water and incubated at room temperature for 5 min. Following the addition of 1 mL of 20% sodium carbonate to the mixture, total polyphenols were determined after 2 hr of incubation at room temperature. The absorbance of the blue color was measured at 765 nm with a spectrophotometer. Quantification was done with respect to the standard curve of Gallic acid. The results were expressed as Gallic acid equivalents (GAE). All determinations were performed in triplicate.

#### 2.5.2. Determination of Anthocyanins

Total anthocyanins were estimated by a pH-differential method [17]. Two dilutions of purple waxy corn extracts were prepared, one with potassium chloride buffer (pH 1.0) and the other with sodium acetate buffer (pH 4.5) diluting each by the previously determined dilution factor. Absorbance was measured simultaneously at 510 and 700 nm after 20 min of incubation at room temperature. The content of total anthocyanins was expressed in mg of cyanidin-3-glucoside equivalents/mg extract using a molar extinction coefficient (*ɛ*) of cyanidin-3-O glucoside of 26900 L mol^−1 ^cm^−1^ and molar weight (MW) (449.2 g mol^−1^).

#### 2.5.3. Determination of Malondialdehyde (MDA)

Level of malondialdehyde (MDA), a relatively stable lipid peroxidation marker, was monitored by using thiobarbituric acid reacting substances (TBARS) assay which is based on MDA reaction with thiobarbituric acid at temperature of 95°C for 60 min to form thiobarbituric acid reactive product. The absorbance of the resultant pink product can be measured at 532 nm. In brief, 100 *μ*L of sample was added to the solution containing 100 *μ*L of 8.1% (w/v) sodium dodecyl sulphate, 750 *μ*L 20% (v/v) acetic acid (pH 3.5), and 750 *μ*L of 0.8% thiobarbituric acid (TBA). The tubes were heated in a water bath at 95°C for one hour and cooled immediately under running tap water. To each tube, 500 *μ*L chilled water and 2500 *μ*L of butanol and pyridine (15 : 1 v/v) were added and the tubes were vortexed and centrifuged at 800 ×g for 20 min. The upper layer was aspirated out and color intensity measured at 532 nm, 1,3,3-tetraethoxypropane (TEP) was used as the reference [18].

#### 2.5.4. Superoxide Dismutase (SOD) Assay

Superoxide dismutase assay was performed using the xanthine/xanthine oxidase reaction as a source of substrate (superoxide) and reduced nitrobluetetrazolium as an indicator of superoxide. The reaction was performed at 25°C. In brief, 20 *μ*L of sample was added to 200 *μ*L of reaction mixture containing 57 mM phosphate buffer solution (KH_2_PO_4_), 0.1 mM EDTA, 10 mM cytochrome C solution, and 50 *μ*M of xanthine solution. Then 20 *μ*L of xanthine oxidase solution (0.90 mU/mL) was added and mixed by gentle inversion. The absorbance was measured at 415 nm [19]. A system devoid of enzyme served as the control and three parallel experiments were conducted.

#### 2.5.5. Catalase (CAT) Assay

Lens catalase activity was determined by colorimetric method, in which lens homogenate was incubated in H_2_O_2_ substrate and the enzymatic reaction. In brief, 10 *μ*L of sample was added to the reaction mixture containing 50 *μ*L of 30 mM hydrogen peroxide (in 50 mM phosphate buffer, pH 7.0), 25 *μ*L of H_2_SO_4_, and 150 *μ*L of KMnO_4_. After mixing thoroughly, the absorbance was measured at 490 nm. A system devoid of the substrate (hydrogen peroxide) was served as the control [20]. The difference in absorbance per unit time was expressed as the activity. One unit was defined as the amount of enzyme required to decompose 1.0 M of hydrogen peroxide per minute at pH 7.0 and 25°C.

#### 2.5.6. Glutathione Peroxidase (GPx) Assay

This assay was performed based on the glutathione recycling method by using 5,5′-dithiobis (2-nitrobenzoic acid) (DTNB) and glutathione reductase. According to this method, the reaction between DTNB and reduced glutathione (GSH) gave rise to the generation of 2-nitro-5-thiobenzoic acid and oxidized glutathione (GSSG). Since 2-nitro-5-thiobenzoic acid was a yellow colored product, GSH concentration could be determined by measuring absorbance at 412 nm. In brief, 20 *μ*L of sample was added to the reaction mixture which contained 10 *μ*L of dithiothreitol (DTT) in 6.67 mM potassium phosphate buffer (pH 7), 100 *μ*L of sodium azide in 6.67 mM potassium phosphate buffer (pH 7), 10 *μ*L of glutathione solution, and 100 *μ*L of hydrogen peroxide. After being mixed thoroughly and incubated at room temperature for 5–10 minutes, 10 *μ*L of DTNB (5,5-dithiobis-2-nitrobenzoic acid) was added and the absorbance at 412 nm s was recorded at 25°C over a period of 5 min [21]. Activities were expressed as nmoles/min/mg lens protein.

#### 2.5.7. Determination of Aldose Reductase (AR) Activity

Aldose reductase activity was determined using spectrophotometric method. The determination was performed by measuring the decrease in NADPH absorbance at 390 nm over a 4-minute period, using DL-glyceraldehyde as a substrate. Each 1.0 mL cuvette containing equal units of enzyme 0.1 M sodium phosphate buffer (pH 6.2) and 0.3 mM NADPH either with or without substrate and inhibitor was prepared. One set of mixtures prepared with an equivalent volume of sodium phosphate buffer instead of tested samples was used as control [22]. Quercetin was used as a positive control of AR inhibitor based on its aldose reductase suppression activity and the anticataractogenesis effect.

### 2.6. Statistical Analysis

All parameters were compared using one-way analysis of variance (ANOVA). The post hoc test was used to identify specific mean differences. They were represented as mean ± standard error mean (mean ± S.E.M). Statistical analysis was carried out using SPSS version 15. Differences were considered significant at *P* value <0.05.

## 3. Results 

### 3.1. Anticataract Effect of Purple Waxy Corn Seeds Extract

**Figure 1:**
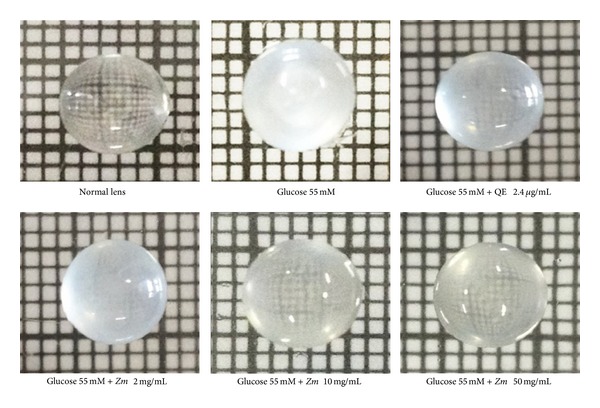
The photograph of male Wistar rat lens in all groups after the 72-hour incubation period.

Table 1 showed that, before the exposure to high concentration of glucose (glucose 55 mM), no significant difference in lens opacity among various groups was observed. The incubation of lens with 55 mM of glucose for 72 hours clearly revealed the increased lens opacity (*P* value <0.001; compared to normal lens) as shown in Figure 1 and Table 1. Interestingly, quercetin and both medium and high doses of purple waxy corn seeds extract could decrease the enhanced lens opacity in model of diabetic cataract induced by high glucose concentration (*P* value <0.001; compared to experimental diabetic cataract group).

### 3.2. Effect of Purple Waxy Corn Seeds Extraction Oxidative Stress Markers

**Figure 2:**
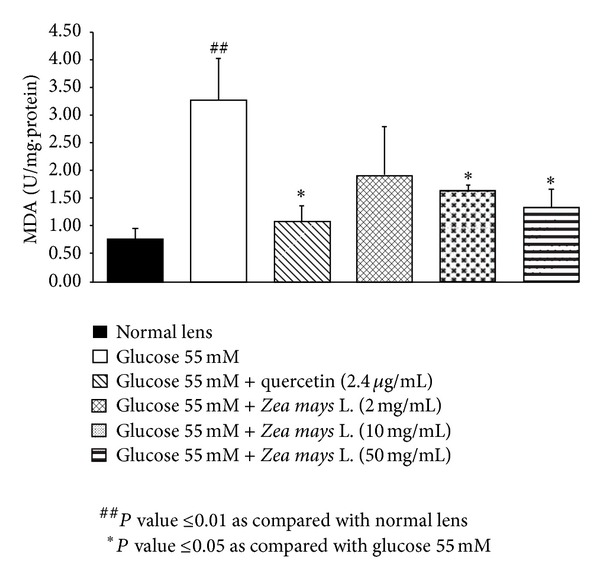
Effect of purple waxy corn seeds extract on MDA level in lens after the 72-hour incubation period. Results are expressed as means ± SEM.

**Figure 3:**
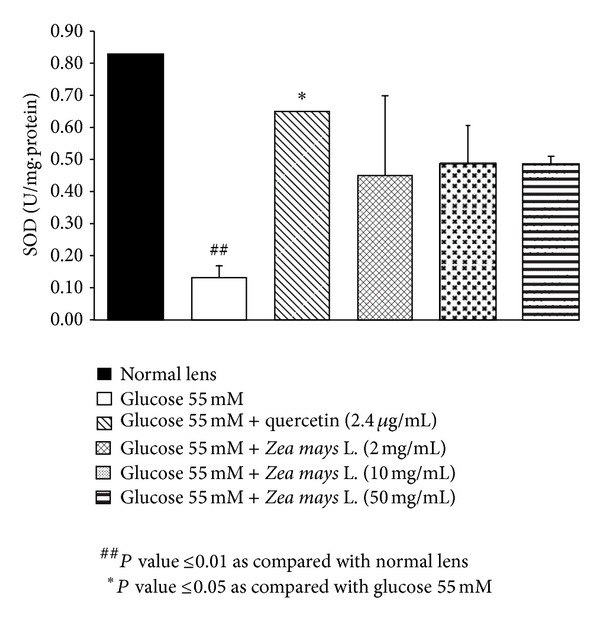
Effect of purple waxy corn seeds extract on SOD activity in lens after the 72-hour incubation period. Results are expressed as means ± SEM.

**Figure 4:**
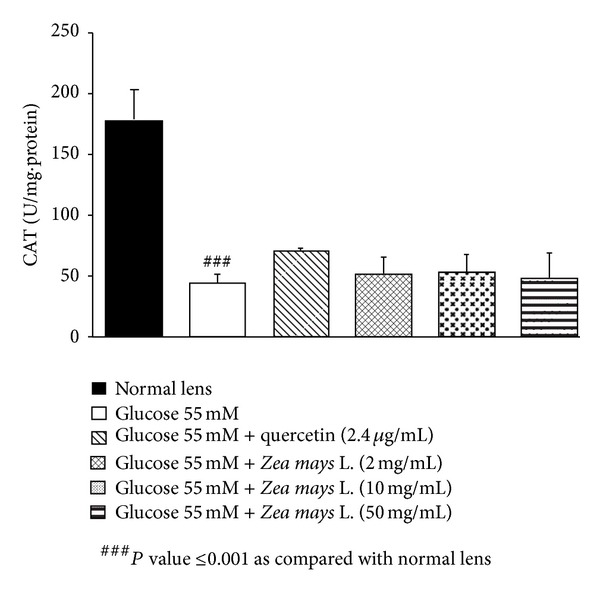
Effect of purple waxy corn seeds extract on CAT activity in lens after the 72-hour incubation period. Results are expressed as means ± SEM.

**Figure 5:**
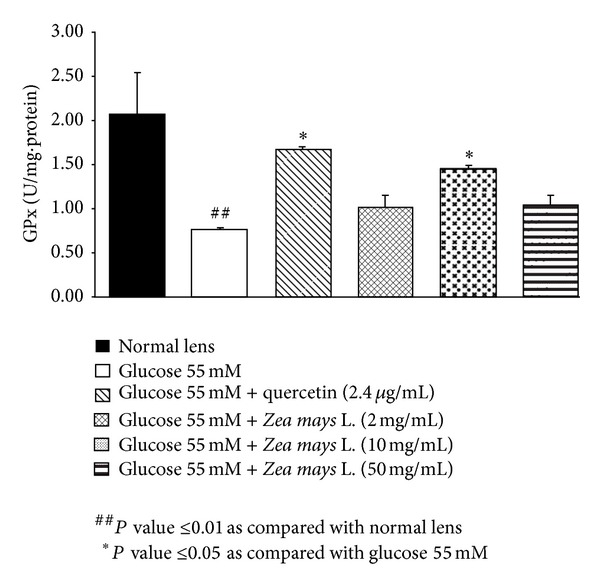
Effect of purple waxy corn seeds extract on GPx activity in lens after the 72-hour incubation period. Results are expressed as means ± SEM.

Based on the previous finding that oxidative stress plays the crucial role on cataractogenesis [23], we also investigated the effect of purple waxy corn seeds extract on oxidative stress markers including level of MDA and the activities of SOD, CAT, and GPx in lens as shown in Figures 2, 3, 4, and 5. Glucose significantly increased MDA level (*P* value <0.01; compared to normal lens) but increased the activities of SOD (*P* value <0.01; compared to normal lens), CAT (*P* value <0.001; compared to normal lens), and GPx (*P* value <0.01; compared to normal lens) in lens. Quercetin, a phenolic compound previously showed anticataractogenesis effect, also mitigated the elevation of MDA level (*P* value <0.05; compared to experimental diabetic cataract group) and the decreased SOD (*P* value <0.05; compared to experimental diabetic cataract group) and GPx (*P* value <0.05; compared to experimental diabetic cataract group) induced by high glucose concentration. However, quercetin failed to show the significant change of CAT activity in the lens. It was found that both medium and high doses of purple waxy corn seeds extract could mitigate the elevation of MDA induced by high glucose exposure which was used to induce the experimental diabetic cataract. Surprisingly, only the medium dose of extract mitigated the decreased GPx activity in lens and no other significant changes were observed.

### 3.3. Effect of Purple Waxy Corn Seeds Extract on Aldose Reductase

**Figure 6:**
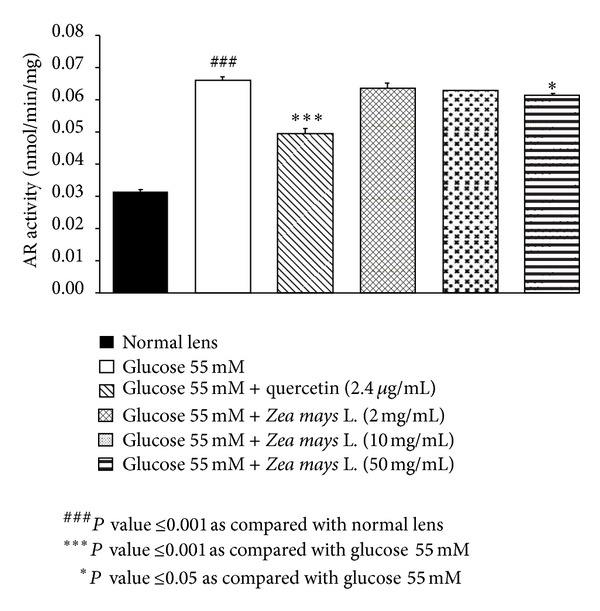
Effect of purple waxy corn seeds extract on AR activity in lens after the 72-hour incubation period. Results are expressed as means ± SEM.

In this study, we also determined the effect of purple waxy corn seeds extract on aldose reductase activity in lens and data were shown in Figure 6. Lens exposed to high glucose concentration or experimental diabetic cataract showed the elevation of aldose reductase (*P* value <0.001; compared to normal lens). Both quercetin and high dose of purple waxy corn seeds extract significantly attenuated the decreased aldose reductase activity induced by high glucose exposure (*P* value <0.001 and 0.05 resp.; compared to experimental diabetic cataract).

## 4. Discussion 

It has been well known that oxidative stress is one of the possible causes of diabetic cataractogenesis. High glucose concentration enhanced the formation of superoxide (O_2_^∙−^) radicals and H_2_O_2_ [15] but decreased antioxidant enzymes such as SOD, CAT, and GPx [5] resulting in the excess oxidative stress reflected by an elevation of lipid peroxidation product or MDA level. The excess oxidative stress was previously reported to induce extensive oxidative modifications on lens proteins especially *α*-crystalline protein, a major protein component of the mammalian eye lens resulting in the enhanced lens opacity [24, 25]. In addition, the markedly elevation of aldose reductase in the lens was also observed. Numerous lines of evidence demonstrated that the AR-mediated intracellular accumulation of polyols induced the collapse and liquefaction of lens fibers, which led to the formation of lens opacities [26, 27].

Recently, considerable attention has been paid to the survey for novel intervention against diabetic cataract. Epidemiologic data have shown that consumption of vitamins, minerals, fiber, and numerous phytochemicals, including flavonoids, can lower the risk of cataract in humans [28]. Phenolic compounds, the potential anticataract agents, could reduce the risk of cataract formation by affecting multiple key pathways pertinent to eye lens opacification, including oxidative stress and polyol pathway. It has been reported that numerous types of phenolic compounds such as quercetin and anthocyanin showed anti-cataract activity [29, 30].

**Figure 7:**
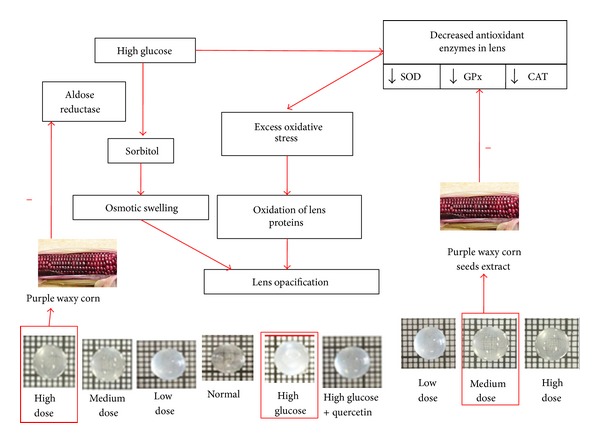
Schematic diagram illustrates possible underlying mechanism of purple waxy corn seeds extract to mitigate lens opacification in experimental diabetic cataractogenesis.

Previous study had revealed that the purple corn contained abundant phenolic compounds such as kaempferol, morin, naringenin, ferulic acid, caffeic acid, quercetin, rutin, and chlorogenic acid [31]. On the basis of beneficial effect of anticataract effect of substances possessing antioxidant and aldose reductase suppression activities, we suggested that the phenolic compounds or the interaction between various compounds presented in the purple waxy corn seeds extract might be responsible for the anticataract effect of the extract. The medium dose of the extract might exert its effect mainly via the increased GPx activity resulting in the decreased oxidative stress reflected by the decreased MDA level in lens. The decreased oxidative stress in turn decreased the oxidation of lens proteins and finally decreased the lens opacification. However, the high dose of the extract appeared to exert the primarily effect via the suppression of aldose reductase resulting in the decreased polyol accumulation in the lens which in turn decreased the swelling of the lens epithelial cells and in the central lens region. This phenomenon then eventually decreased the degeneration of lenticular fibers and lens opacity [32] as shown in Figure 7. As the degeneration of lenticular fibers progressed, the entire cortex became opaque and the formation of nuclear opacity occurred.

## 5. Conclusion

The present results suggest that purple waxy corn is the potential beneficial food to protect against diabetic cataract. Its underlying mechanism appears to depend on dose. Medium dose of the seeds extract exerts its effect via the decreased oxidative stress whereas the high dose of the seeds extract exerts its effect via the suppression effect of aldose reductase, the rate limiting enzyme in polyol pathway. However, further researches in vivo and the searching for active ingredient are still necessary.

## Figures and Tables

**Table 1:** Effect of purple waxy corn seeds extract on lens opacity in normal and experimental groups before and after the 72-hour incubation period.

Groups	Transparent squares (before incubation)	Transparent squares (after incubation)	*P* value
Gr. 1: normal lens (artificial aqueous humor, pH = 7.8)	151.00 ± 10.00	140.00 ± 2.00	
Gr. 2: glucose 55 mM	141.50 ± 3.50	0.50 ± 0.50^###^	0.000
Gr. 3: glucose 55 mM + quercetin 2.4 *µ*g/mL	152.50 ± 1.50	77.00 ± 2.00***	0.000
Gr. 4: glucose 55 mM + *Z. mays* 2 mg/mL	141.50 ± 3.50	14.00 ± 2.00	0.069
Gr. 5: glucose 55 mM + *Z. mays* 10 mg/mL	149.50 ± 6.50	71.50 ± 2.50***	0.000
Gr. 6: glucose 55 mM + *Z. mays* 50 mg/mL	138.50 ± 3.50	86.00 ± 1.00***	0.000

^###^ *P* value <0.001 when compared to normal lens.

***P value <0.001 when compared to diabetic cataract (glucose 55 mM).
